# Naturalized *Escherichia coli* in Wastewater and the Co-evolution of Bacterial Resistance to Water Treatment and Antibiotics

**DOI:** 10.3389/fmicb.2022.810312

**Published:** 2022-05-30

**Authors:** Daniel Yu, Kanghee Ryu, Shuai Zhi, Simon J. G. Otto, Norman F. Neumann

**Affiliations:** ^1^School of Public Health, University of Alberta, Edmonton, AB, Canada; ^2^Antimicrobial Resistance – One Health Consortium, Calgary, AB, Canada; ^3^School of Medicine, Ningbo University, Ningbo, China; ^4^The Affiliated Hospital of Medical School, Ningbo University, Ningbo, China; ^5^Human-Environment-Animal Transdisciplinary Antimicrobial Resistance Research Group, School of Public Health, University of Alberta, Edmonton, AB, Canada; ^6^Healthy Environments, Centre for Health Communities, School of Public Health, University of Alberta, Edmonton, AB, Canada

**Keywords:** antibiotic resistance, water treatment resistance, genome, wastewater, naturalized *Escherichia coli*

## Abstract

Antibiotic resistance represents one of the most pressing concerns facing public health today. While the current antibiotic resistance crisis has been driven primarily by the anthropogenic overuse of antibiotics in human and animal health, recent efforts have revealed several important environmental dimensions underlying this public health issue. Antibiotic resistant (AR) microbes, AR genes, and antibiotics have all been found widespread in natural environments, reflecting the ancient origins of this phenomenon. In addition, modern societal advancements in sanitation engineering (i.e., sewage treatment) have also contributed to the dissemination of resistance, and concerningly, may also be promoting the evolution of resistance to water treatment. This is reflected in the recent characterization of naturalized wastewater strains of *Escherichia coli*—strains that appear to be adapted to live in wastewater (and meat packing plants). These strains carry a plethora of stress-resistance genes against common treatment processes, such as chlorination, heat, UV light, and advanced oxidation, mechanisms which potentially facilitate their survival during sewage treatment. These strains also carry an abundance of common antibiotic resistance genes, and evidence suggests that resistance to some antibiotics is linked to resistance to treatment (e.g., tetracycline resistance and chlorine resistance). As such, these naturalized *E. coli* populations may be co-evolving resistance against both antibiotics and water treatment. Recently, extraintestinal pathogenic strains of *E. coli* (ExPEC) have also been shown to exhibit phenotypic resistance to water treatment, seemingly associated with the presence of various shared genetic elements with naturalized wastewater *E. coli*. Consequently, some pathogenic microbes may also be evolving resistance to the two most important public health interventions for controlling infectious disease in modern society—antibiotic therapy and water treatment.

## Introduction

The world is currently in an antibiotic resistance crisis. The severity of this crisis is best exemplified by bacterial pathogens of pandemic concern, including the multidrug-resistant and extremely drug-resistant *Mycobacterium tuberculosis*, the extended-spectrum beta-lactamase (ESBL) and carbapenem-resistant Enterobacteriaceae (CRE), as well as several microbes collectively known as the “ESKAPE” pathogens, including vancomycin-resistant *Enterococcus faecium* (VRE), methicillin-resistant *Staphylococcus aureus* (MRSA), *Klebsiella pneumoniae*, *Acinetobacter baumannii*, *Pseudomonas aeruginosa*, and *Enterobacter* spp. (Davies and Davies, 2010; Ventola, 2015; Tacconelli et al., 2018; Jernigan et al., 2020). The rapid growth of antibiotic resistance has placed an incredible burden on the health system (US Department of Health and Human Services and CDC, 2019; Serra-Burriel et al., 2020; Nelson et al., 2021) and without urgent action, estimates suggest that drug resistant infections will cost $100 trillion dollars (USD) and cause the deaths of 10 million people annually by 2050 (O’Neill, 2016). Indeed, the world appears to be headed toward a “post-antibiotic era” (Kwon and Powderly, 2021), where many infectious diseases may become effectively untreatable and routine medical procedures too dangerous to perform without robust anti-infection preventative therapies.

With the looming threat of a post-antibiotic era, increased efforts have been redirected toward identifying and mitigating the major drivers of antibiotic resistance. While the evolution and transmission of antibiotic resistance have been particularly well-characterized within human (Shallcross, 2014; Center for Disease Dynamics, Economics and Policy, 2015; Ventola, 2015; Holmes et al., 2016; Pal et al., 2016) and animal contexts (Cabello et al., 2013; Center for Disease Dynamics, Economics and Policy, 2015; Martin et al., 2015; Holmes et al., 2016; Pal et al., 2016), the role of the environment in the current antibiotic resistance crisis has received considerably less attention (McCubbin et al., 2021). The focus on human/animal health is understandable and justifiable given that acquired resistance is largely driven by anthropogenic use of antimicrobials (Skurnik et al., 2006; Grall et al., 2015), but recent efforts have been made to address the environmental dimensions of the evolution and dissemination of antibiotic resistance (Martinez, 2009; Wright, 2010; Finley et al., 2013; Perry and Wright, 2013; Berendonk et al., 2015; Bengtsson-Palme et al., 2018; Stanton et al., 2020). Nevertheless, most One Health approaches designed to combat antibiotic resistance continue to focus primarily on mitigating antibiotic resistance-promoting activities and promoting stewardship in human, agricultural, and aquacultural sectors (Hoelzer et al., 2017; Tang et al., 2017; Scott et al., 2018; Nathwani et al., 2019).

Despite the under-representation of environmental health perspectives in One Health approaches to antibiotic resistance (Iossa and White, 2018; McCubbin et al., 2021), both natural and man-made environments closely interface with the cycling of antibiotic resistance across human and animal sectors. Although this is most evident in “antibiotic pollution” of the environment due to anthropogenic use of antibiotics (Kraemer et al., 2019), the natural environment also serves as an important reservoir of antibiotic resistant microbes and their resistance genes (Huijbers et al., 2015). In particular, “naturalized” microbial populations—microbes that have evolved to primarily inhabit non-host environments (Ishii et al., 2006; Devane et al., 2020)—may play an important role in the origin, evolution, and dissemination of antibiotic resistance. Reflecting this, sewage/wastewater treatment plants, have been identified as an important “hotspot” for antibiotic resistance, and in this review, we examine the natural wastewater microbiome as a potential reservoir for the emergence of antibiotic resistance in the microbial world. As a model of our understanding, we describe the evolutionary emergence of naturalized wastewater *Escherichia coli*. These strains of *E. coli* appear to have evolved to live and survive in modern-day, engineered wastewater treatment plants. They display a certain level of resistance to wastewater treatment (i.e., conventional treatment as well as disinfection; chlorination, advanced oxidants, and heat) and carry an abundance of antibiotic resistance genes (Zhi et al., 2017, 2019), implicating their role as a potential reservoir for both antibiotic and wastewater treatment resistance. Similarly, some pathotypes of *E. coli*, such as the extra-intestinal pathogenic *E. coli* (ExPEC) also appear to resist wastewater treatment and exhibit similar genetic properties as wastewater naturalized strains (Zhi et al., 2020). Concerningly, the evidence suggests that microbes may have evolved adaptations against two critical public health interventions that are fundamental for the control of infectious disease in modern society—water treatment/sanitation and antibiotic therapy.

## Environmental Origins of Antibiotic Resistance: The Role of Natural Microbial Communities

It is undeniable that, since the “golden era” of antibiotic discovery (Aminov, 2010), the widespread use of antibiotics in clinical and agricultural contexts have primarily driven the current antibiotic resistance crisis (Shallcross, 2014; Martin et al., 2015). However, it is important to note that the current crisis merely represents a relatively recent development in the long history of antibiotic resistance in the microbial world. Indeed, the anthropogenic adoption of antibiotics has simply exerted an extreme global selective pressure promoting the emergence and dissemination of antibiotic resistance in microbes at an unprecedented scale.

Considering this, antibiotic resistance can be thought of as either intrinsic, which refers to the basal level of resistance provided by the inherent structural or biochemical characteristics of the bacterial cell prior to the widespread use of antibiotics, or acquired, which mainly refers to the recent transfer of genetic determinants conferring resistance to bacterial populations, particularly pathogens, and in response to the selective pressure exerted by the rise in antibiotic use (Martinez, 2009). The concept of intrinsic resistance directly implicates the environment in the evolution of antibiotic resistance, especially considering that microbial communities have inhabited natural environments for billions of years before the first appearance of humans or animals. Reflecting this, it is now recognized that various antibiotic resistance determinants have ancient, often environmental, origins (Mindlin et al., 2008; Martinez, 2009; D’Costa et al., 2011; Perron et al., 2015).

Antibiotic resistance is fundamentally a natural phenomenon, and this is particularly apparent in naturalized microbial communities. Microbial populations that are “naturalized” refer to those subgroups that have evolved to preferentially survive, inhabit, and grow in external, non-host environments as their primary niche (Ishii et al., 2006; Devane et al., 2020). Some naturalized microbes belong to host-associated taxa, but appear to have diverged from their host-derived counterparts very recently, or in some cases millions of years ago (Walk et al., 2009; Staley et al., 2014; Walk, 2015), and include organisms such as *E. coli* (Ishii et al., 2006; Jang et al., 2017). Reflecting their evolutionary divergence, these naturalized populations have been found to be genotypically distinct from host-associated lineages (Ishii et al., 2006; Tymensen et al., 2015; Zhi et al., 2019), and appear to have evolved various adaptive mechanisms to thrive outside of the host niche. Importantly, this may include adaptive mechanisms associated with antibiotic resistance, as antibiotics present in the environment play important roles in the dynamics and composition of these microbial populations (Gao et al., 2020).

The connection between natural microbes and antibiotic resistance is immediately evident in their role as producers of antibiotic compounds. Many of the major classes of antibiotics—including those under considerable threat in the current resistance crisis—originated in microbes naturally occurring in the environment, particularly those inhabiting soil environments (Chandra and Kumar, 2017). The antibiotic potential of naturally-produced bioactive molecules was recognized early during antibiotic discovery, such as seen in the discovery of penicillin by Fleming from the soil-derived *Penicillium* mold (Fleming, 1929). Recognizing the wealth of potential antibiotic compounds present in soil microbial communities, Waksman pioneered comprehensive screening protocols on the soil dwelling filamentous actinomycetes, which led to the discovery of antibiotics such as streptomycin and neomycin, the first antibiotic active agents against tuberculosis (Schatz et al., 1944; Waksman and Lechevalier, 1949). Waksman’s work not only established actinomycetes as prolific antibiotic producers, but also specifically identified the genus *Streptomyces* as an important producer of antibacterial products (Waksman et al., 2010). Reflecting this, actinomycetes and streptomycetes have been found to possess remarkably large genomes, and typically harbor genomic regions specifically dedicated to the biosynthesis of various natural products (Traxler and Kolter, 2015), demonstrating the vast antibiotic producing potential of natural, and specifically soil-dwelling, microbes. Indeed, the majority of antibiotics that have been discovered since the “golden era” have either been characterized as, or were derived from, natural products produced primarily by actinomycetes, as well as other environmental bacteria or environmental fungi (Hutchings et al., 2019).

As antibiotics serve inhibitory, antibacterial purposes in clinical and laboratory settings, it was immediately assumed that these bioactive products would also mediate inter-species competition and “microbial warfare” in natural environments (Allen et al., 2010), which would in turn select for the evolution of resistance against these bioactive compounds. Although the antibacterial effects of some natural microbial products have been demonstrated (Currie et al., 1999; Neeno-Eckwall et al., 2001), naturally-produced antibiotic compounds are typically produced and released at concentrations significantly lower than the minimum inhibitory concentrations typically used in clinical medicine. Furthermore, as the *in situ* physiologic concentrations of naturally-produced antibiotic compounds have yet to be consistently measured, the antibacterial functions attributed to these compounds in natural contexts remain unclear. Despite this, it is well understood that sub-inhibitory concentrations of antibiotics still generate genotypic and phenotypic variability upon which selection can act (López and Blázquez, 2009; Laureti et al., 2013), which can in turn promote the development of antibiotic resistance in natural microbial communities (Andersson and Hughes, 2014; Murray et al., 2018).

Alternative functions for the bioactive small molecules produced by natural microbial communities have been proposed. In particular, a growing body of evidence indicates that many natural microbial products exhibit hormesis (Davies et al., 2006), in which they exhibit a dose-dependent antibiotic effect at high concentrations but appear to play distinct functions at *in situ* concentrations present in natural environments. For example, at lower concentrations, naturally-produced bioactive molecules appear to activate intercellular signaling pathways important in microbial community dynamics, particularly in modulating gene expression related to quorum sensing, biofilm formation, adherence, and virulence (Traxler and Kolter, 2015; Murray et al., 2018). Reflecting this, some of the earliest antibiotic substances isolated from *P. aeruginosa* were later found to consist of quorum-sensing molecules that simultaneously displayed potent antibacterial activities (Kaufmann et al., 2005; Dubern and Diggle, 2008). As such, natural bioactive compounds may be involved in metabolic signaling networks in microbial communities, whereby the receptors for these molecules involve the same cellular structures that are targeted for inhibition during antibiotic use (Allen et al., 2010). Recently, an alternative function for naturally-produced antibiotic compounds in predation dynamics have been proposed, as it has been suggested that antibiotic production may be the result of predation-driven selection during nutritional and evolutionary bottlenecks (Leisner et al., 2016).

The finding that antibiotics appear to mediate other functions beyond exerting antibacterial effects in natural environments suggests that they were distributed throughout the environment long before their introduction for therapeutic and prophylactic purposes. Indeed, resistance-conferring genes and mutations have been shown to impact metabolism, allowing particular strains to utilize various alternative peptide, phosphorous, and carbon sources for nutrition (Allen et al., 2010). As such, the same evolutionary events that confer resistance in clinical or laboratory contexts may alternatively promote metabolic flexibility in the environment. Similarly, extensive gene knockout analyses have revealed a myriad of genes that, while primarily involved in other functions, contribute to an antibiotic resistance phenotype (Gomez and Neyfakh, 2006; Breidenstein et al., 2008; Duo et al., 2008; Tamae et al., 2008). Conversely, studies have shown that microbes can carry a plethora of resistance genes, but concomitantly, are susceptible to antibiotic treatment (Zhi et al., 2019), suggesting that these genes may provide other important functions within the life history of a microbe. Considering the prevalence of antibiotic resistance genes in the microbial world, particularly in environmental microbial communities, resistance determinants have likely evolved in response to a variety of selection pressures prior to the discovery and widespread use of antibiotics as therapeutics.

Consequently, microbial populations found in environmental matrices appear to demonstrate a basal level of resistance against a single antibiotic or a group of antibiotics. For instance, a comprehensive resistance screen of morphologically distinct spore-forming actinomycetes against 21 antibiotics conducted by D’Costa et al. (2006) identified several strains that were resistant to an average of 7 or 8 antibiotics across the major classes, indicating that these strains were naturally multidrug resistant. Naturally-resistant microorganisms within these microbial communities may then serve as reservoirs of antibiotic resistance, and resistance-adjacent, genes, especially if they become incorporated into mobile genetic elements that can be horizontally acquired by microbes of clinical concern. Reflecting this, Forsberg et al. (2012) characterized soil bacteria that shared a remarkably similar antibiotic resistome compared to various human pathogens. Screening of the multidrug-resistant soil bacteria revealed several resistance cassettes against antibiotics including beta-lactams, aminoglycosides, amphenicols, sulfonamides, and tetracyclines, containing genes with 100% identity to those found in various human pathogens. As the sequence similarity encompassed mobilization sequences, the resistance elements in question may have been laterally transferred to the pathogenic microbes. Importantly, the antibiotic resistome of environmental microbial communities extends beyond known resistance genes and may include novel resistance determinants that have yet to be characterized. Indeed, comprehensive screens of resistance genes and mobile genetic elements in environmental, human, and animal microbiomes have revealed relatively low abundances of known antibiotic resistance elements in natural environments, but the high taxonomic and genetic diversity identified in environmental microbiomes indicate that they may harbor a vast collection of currently unknown resistance genes that could be transferred to pathogens (Pal et al., 2016; Steen et al., 2019).

Overall, there remains relatively little evidence for the *direct* transfer of antibiotic resistance genes between the environmental resistome and clinical pathogens. Although there is great potential for the horizontal transfer of resistance genes, transfer events are only made evident when the genes become clinically relevant. As such, while several transfer events may occur, only a select few of the transferred genes will become fixed in a pathogenic population as natural selection will simultaneously act at the level of the gene itself and on whether the gene is maintained following the transfer event. Nevertheless, certain antibiotic resistance genes, especially those of particular concern in this antibiotic resistance crisis, appear to have origins in environmental microbes. One such class of antibiotic resistance genes include the beta-lactamases, which are estimated to have originated over 2 billion years ago (Hall and Barlow, 2004). Interestingly, several beta-lactamase families have been estimated to have mobilized from bacterial chromosomes onto plasmids millions of years ago (Barlow and Hall, 2002). For instance, despite their recent emergence and proliferation within clinical pathogens, certain beta-lactamases within the OXA family appear to have originated from the free-living environmental *Shewanella* species. Indeed, the beta-lactamase OXA-48, which mediates carbapenem resistance in Enterobacteriaceae, appears to have originated from the chromosomal *bla*_OXA-54_ gene found in *Shewanella oneidensis*, which is commonly found in lake sediments (Poirel et al., 2004). Similarly, the beta-lactamase OXA-181 appears to have originated from chromosomal genes carried by *Shewanella xiamenensis*, an environmental microbe commonly found in marine and freshwater environments (Potron et al., 2011). This also appears to be the case for class A beta-lactamases, which can hydrolyze a wide range of extended spectrum cephalosporins and monobactams, rendering many antibiotics ineffective (Cantón et al., 2012). While there are several families of class A extended spectrum beta-lactamases, the CTX-M family has received particular attention due to its explosive dissemination worldwide in a “CTX-M pandemic” (Cantón and Coque, 2006). Interestingly, several CTX-M beta-lactamase clusters appear to have been mobilized from chromosomal beta-lactamase genes harbored by various free-living *Kluyvera* species that are ubiquitous in soil, water, and sewage environments (Watson et al., 2017). Indeed, the chromosomal beta-lactamase gene *kluC* found in *Kluyvera cryocrescens* is thought to be the ancestor to the CTX-M-1 cluster (Decousser et al., 2001) and *kluA* from *K. ascorbata* may have given rise to the CTX-M-2 cluster (Humeniuk et al., 2002), while the genes *kluG*, *kluY*, and *bla_CTX-M-78_* from *K. georgiana* appear to be the likely origin of the CTX-M-8 (Poirel et al., 2002), CTX-M-9 (Olson et al., 2005), and CTX-M-25 (Rodríguez et al., 2010) clusters, respectively.

*Kluyvera* species have also been implicated in the origin of other resistance genes, such as the fosfomycin-resistance gene *fosA4* (Rodriguez et al., 2018). A similar environmental origin has been proposed for the *qnr* family of genes that confer resistance to quinolones. Indeed, *qnr*-like genes have been characterized in environmental microbes such as waterborne Vibrionaceae (Poirel et al., 2005a) and the marine and freshwater-dwelling *Shewenella algae* (Poirel et al., 2005b). Interestingly, when these environmental *qnr* genes were transformed into *E. coli*, they conferred resistance to quinolone antibiotics (Poirel et al., 2005a). As this evidence indicates, although they may not directly pose a risk to the health of humans and animals, microorganisms of environmental origin, including natural and recently naturalized microbes, may act as important reservoirs for the mobilization of key antibiotic resistance genes that, when acquired by pathogenic populations, can render antibiotic therapies ineffective.

## Wastewater Treatment Plants as “Hotspots” for Antibiotic Resistance

### Dissemination of Antibiotic Resistance From Wastewater Treatment Plants

**Figure 1:**
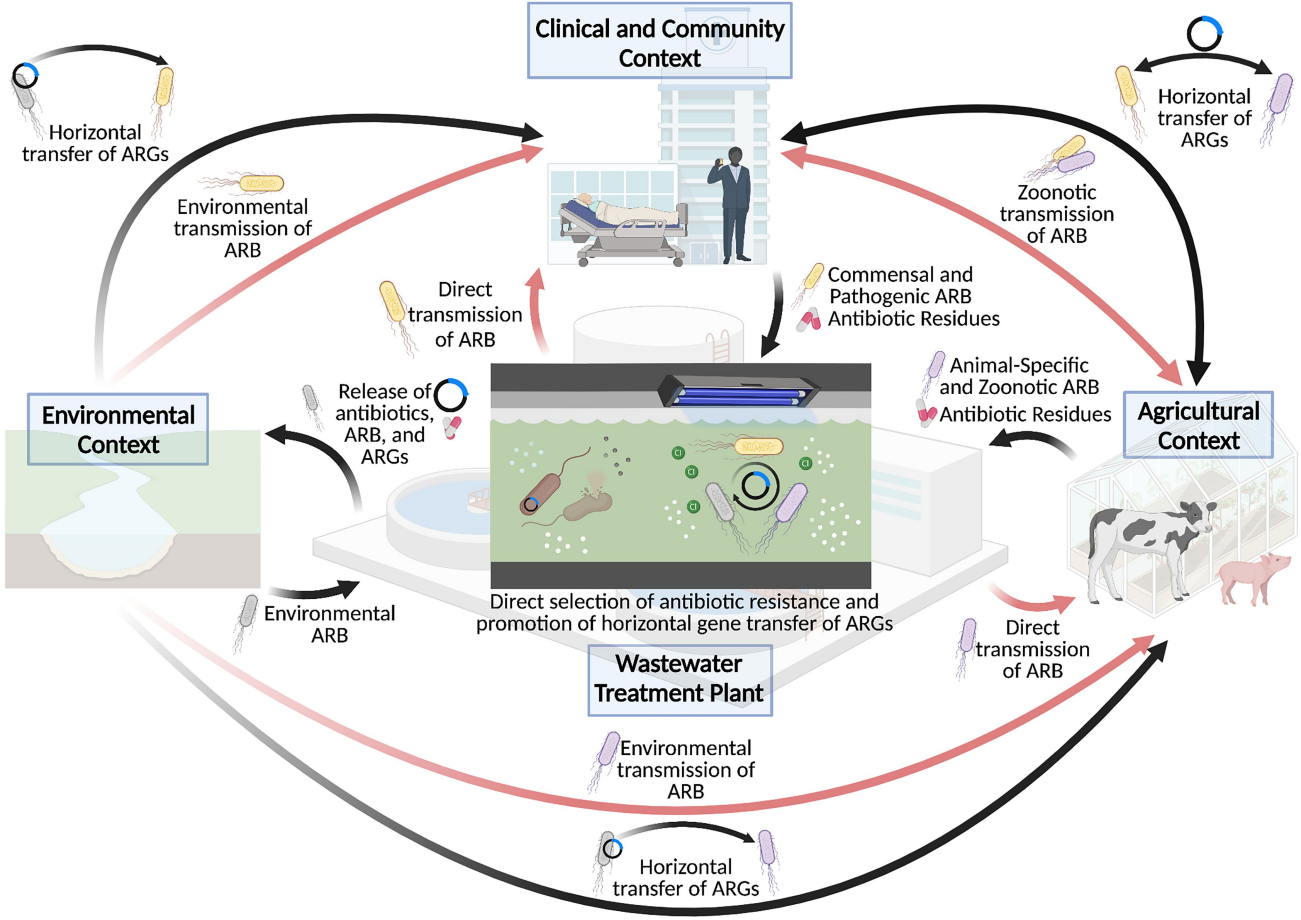
Wastewater treatment plants as a hotspot for the dissemination and selection of antibiotic resistance. Wastewater treatment plants have played an important role in driving the antibiotic resistance crisis, particularly through cycling antibiotics, antibiotic resistant bacteria (ARB) and antibiotic resistance genes (ARG) between clinical, agricultural, and environmental sectors, as depicted by the black arrows. This in turn contributes to the transmission of antibiotic resistant bacteria to human and animal hosts, as shown in the red arrows. Importantly, the conditions of the wastewater treatment plant may also directly contribute to the selection of antibiotic resistance or promote the mobilization of resistance genes within the wastewater matrix, which further exacerbates the antibiotic resistance crisis. Created with BioRender.com.

A better understanding of the environmental influences on the rapid growth of antibiotic resistance has led to the identification of environmental hotspots in the current antibiotic resistance crisis. In particular, wastewater treatment plants have received considerable attention as important drivers of antibiotic resistance, especially as a conduit for the dissemination of antibiotic resistance from anthropogenic activities to natural environments (Figure 1). Indeed, antibiotics, antibiotic resistant microbes, and their associated resistance genes are widespread in wastewater matrices (Schwartz et al., 2003; Baquero et al., 2008), such that their subsequent release *via* effluent discharge represents an important source of antibiotic pollution in the surrounding environment. As such, despite the recent technological emergence of wastewater treatment plants to manage the sanitary wastes of densely populated societies (i.e., within last century), wastewater treatment plants have been identified as important “hotspots” in the dissemination of antibiotic resistance (Michael et al., 2013; Rizzo et al., 2013).

Although water treatment leverages a variety of processes to eliminate the various microbiological, chemical, and physical contaminants prevalent in wastewater, conventional treatment regimens do not reliably eliminate all antibiotics and other pharmaceutical compounds from the wastewater matrix (Michael et al., 2013). Biological processes, for instance, which include sorption to activated sludge and microbiological degradation, appear to be quite variable in their capacity to eliminate different classes of antibiotics. While several studies have demonstrated the efficient removal of various antibiotics through biological means, including beta-lactams (Watkinson et al., 2009), tetracyclines (Yang et al., 2005), sulfonamides (Yang et al., 2005; Li et al., 2009), and quinolones (Zorita et al., 2009), removal efficiencies overall vary widely across studies and water treatment processes. Indeed, other studies have demonstrated an incomplete removal of the same antibiotics in response to biological processes (Brown et al., 2006; Cha et al., 2006), whereas other antibiotic classes such as macrolides appear to be recalcitrant to removal through biological treatment (Li and Zhang, 2011). Studies evaluating the removal efficiencies of oxidative and disinfection treatments also yielded varied results. Ozonation, for instance, appears to remove a significant degree of several antibiotic classes, though the exact efficiency depended on the specific antibiotic (Michael et al., 2013). Similarly, chlorination has been demonstrated to remove certain antibiotics such as cephalexin with high efficiency, while exhibiting incomplete removal of various other compounds including the human-derived erythromycin metabolite erythromycin-H_2_O, as well as antibiotics such as sulfamethoxazole and trimethoprim (Li and Zhang, 2011). Moreover, while ultraviolet (UV) irradiation has been shown to remove antibiotics including sulfonamides and quinolones at high efficiencies, macrolides and tetracycline were largely resilient and were removed at significantly lower rates (Kim et al., 2009).

Ultimately, although different treatment processes may collectively contribute to the efficient removal of each antibiotic class, the wide range observed in the removal efficiencies of each antibiotic in response to different treatment process, and the variability in treatment programs used across wastewater treatment plants, indicates that water treatment overall does not completely remove antibiotics from the wastewater matrix. Reflecting this, antibiotics have been increasingly identified in treated wastewater effluents (Zhang and Li, 2011; Östman et al., 2017; Rodriguez-Mozaz et al., 2020) and appear to be particularly high in effluents from the pharmaceutical industry and hospitals, and which have been shown to contribute to hotspots of antibiotic resistance in natural environments (Karkman et al., 2019). Furthermore, while antibiotics themselves appear to be widespread in wastewater matrices, research on the fate of antibiotics during water treatment do not typically address the antibiotic metabolites that are produced following human and animal use (Michael et al., 2013). As such, the selection potential of discharged effluents may be greater than current estimates suggest. Given that current policies surrounding antibiotic resistance and antibiotic pollution do not address the impact of antibiotics present in treated effluents, wastewater treatment plants will continue to represent an important source of antibiotic pollution promoting the selection of antibiotic resistance in natural environments (Barancheshme and Munir, 2018; Kraemer et al., 2019).

Beyond antibiotics, wastewater treatment plants are also implicated in the large-scale release of antibiotic resistance genes into downstream environments. The removal efficiency for resistance genes during water treatment is similarly variable across treatment processes and water treatment programs (Wang and Chen, 2020). For instance, while some studies demonstrated that biological membrane processes could efficiently remove tetracycline (*tetW* and *tetO*) and sulfonamide (*sul*1) resistance genes (Li and Zhang, 2011), others found that biological processes such as biological filter and anaerobic techniques did not significantly reduce any resistance genes during water treatment (Yuan et al., 2016). Furthermore, while ozonation was found to reliably reduce antibiotic resistance genes, disinfection processes, such as chlorination and UV irradiation, were found to be comparatively inefficient, exhibiting negligible removal rates for various resistance genes (McKinney and Pruden, 2012; Stange et al., 2019). Indeed, the finding that certain resistance genes have even been enriched following water treatment (Laht et al., 2014; Bengtsson-Palme et al., 2016) suggests that, like antibiotics, the associated resistance genes may also persist during water treatment. In a survey of antibiotic resistance genes across wastewater samples, Szczepanowski et al. (2009) identified 123 clinically-relevant resistance genes in the final effluents. Similarly, Mao et al. (2015) identified several genes conferring resistance against tetracycline, sulfonamides, quinolones, and erythromycin that were discharged at higher rates in the final effluents than their initial prevalence in influents prior to treatment.

The wide prevalence of antibiotic resistance genes in wastewater raises the possibility of discharged effluents introducing resistance genes to downstream environmental microbial communities. This prospect is particularly concerning considering that these resistance genes may be mobile (Davies and Davies, 2010). For instance, using a high-throughput array to quantify antibiotic resistance genes and their mobilization potential during wastewater treatment, Karkman et al. (2016) found that water treatment enriched various genes involved in gene mobilization, including a *Tn25*-type transposase gene and various clinical class 1 integrons—elements that are implicated in the evolution and dissemination of antibiotic resistance (Babakhani and Oloomi, 2018; Souque et al., 2021). Similarly, using a metagenomic approach, Che et al. (2019) identified a wide prevalence of antibiotic resistance genes conferring resistance to aminoglycosides, beta-lactams, tetracycline, sulfonamides, chloramphenicol, trimethoprim, and quinolones in wastewater effluents, of which over half appeared to be harbored on mobile genetic elements such as plasmids and integrative and conjugative elements. As multi-drug resistant plasmids related to environmental bacteria have also been identified in wastewater effluents (Szczepanowski et al., 2004), wastewater discharge may introduce and promote the horizontal exchange of resistance genes with naturalized microbial communities in environments downstream of wastewater treatment plants.

### Selection of Antibiotic Resistance Within Wastewater Treatment Plants

The wide range of antibiotic resistance genes and mobile genetic elements that are released following water treatment points to the presence of an extensive and highly mobile resistome in wastewater. With such an accessible reservoir of mobile antibiotic resistance genes, the importance of wastewater treatment plants in the antibiotic resistance crisis may extend well beyond the dissemination of antibiotic resistant-determining contaminants in downstream environments. In fact, the wastewater matrix itself may be particularly hospitable for the acquisition and dissemination of antibiotic resistance.

Effectively representing an “endpoint” in the urban water cycle, wastewater treatment plants collect antibiotic-impacted waste from various human, animal, and environmental sources prior to treatment. As a result, wastewater matrices constitute a complex mix of environmental, clinical, and agricultural microorganisms, infused in a concoction of toxic contaminants including biocides, heavy metals, toxic organics, antibiotics, and other pharmaceuticals (Östman et al., 2017). Concerningly, antibiotic resistant bacteria themselves are major constituents of wastewater microbiomes (Fouz et al., 2020). A wide range of antibiotic resistant microbes, including clinically relevant populations such as ESBL-producing Enterobacteriaceae (Amador et al., 2015; Galler et al., 2018; Scaccia et al., 2020; Zhi et al., 2020), vancomycin-resistant *Enterococcus* (Iweriebor et al., 2015; Nishiyama et al., 2015; Galler et al., 2018; Gouliouris et al., 2019), and MRSA (Galler et al., 2018), appear to be widespread in wastewater matrices, representing a potential source of antibiotic resistant bacteria that can be released into downstream environments through effluent discharge.

Beyond this resistant subpopulation, however, the wastewater matrix may also be particularly conducive for the evolution and transmission of antibiotic resistance within the wastewater treatment plant itself. Wastewater matrices comprise a particularly nutrient-rich environment that can support a complex microbiome of human, animal, and environmental microbes. Considering that this microbiome is accompanied by an extensive, highly mobile resistome (Schlüter et al., 2007; Popowska and Krawczyk-Balska, 2013; Ju et al., 2019; Rodríguez et al., 2021) of clinically relevant and novel environment resistance genes, various selection pressures can act in parallel to promote the development of resistance in microbes present in the wastewater matrix. Intuitively, the high concentrations of antibiotics in wastewaters, particularly from pharmaceutical industries (Larsson et al., 2007; Larsson, 2014; Bengtsson-Palme and Larsson, 2016), can strongly select for antibiotic resistance within the wastewater matrix. Outside of this context, however, the concentrations of antibiotics in wastewater treatment plants do not typically exceed the minimum inhibitory concentrations that define resistance in clinical and laboratory settings (Szczepanowski et al., 2009; Ju et al., 2019). Despite this, sub-inhibitory concentrations of antibiotics can still select for resistance, and they appear to be a particularly important driver in doing so (Gullberg et al., 2011; Marshall and Levy, 2011; Andersson and Hughes, 2012; Bengtsson-Palme and Larsson, 2016). As an example, various antibiotics, even at sub-lethal doses, have been shown to activate the bacterial SOS response (Miller et al., 2004; Mesak et al., 2008; Baharoglu and Mazel, 2011), which can generate genetic diversity upon which selection can act and promote the development of adaptive mechanisms (Podlesek and Žgur Bertok, 2020). Although the SOS response does not necessarily accelerate the evolution of antibiotic resistance, it does appear to mediate adaptation by increasing bacterial fitness to the presence of sub-inhibitory concentrations of antibiotics (Torres-Barceló et al., 2015). Furthermore, the SOS response has been linked to increased rates of gene mobilization (Beaber et al., 2004; Guerin et al., 2009), which may further promote the dissemination of antibiotic resistance across microbial populations. As studies have demonstrated that wastewater microbial communities can undergo an extensive degree of mobile genetic exchange, including across phyla (Li et al., 2018), the presence of antibiotics in wastewater appears to exert a strong selective pressure for the dissemination of antibiotic resistance within the wastewater microbiome (Figure 1).

Beyond direct selection, the wastewater matrix and the treatment processes themselves provide other selective pressures that can simultaneously promote the development of antibiotic resistance (Figure 1). The high concentrations of biocides and heavy metals in wastewater matrices (Östman et al., 2017), for instance, have been implicated in the co-evolution of antibiotic resistance alongside metal resistance (Pal et al., 2015; Wales and Davies, 2015). This can occur through the co-localization of biocide- and metal-resistance genes with antibiotic resistance genes on the same genetic elements, such that the selection of one resistance gene will lead to the selection of the whole genetic element. While the previous studies have characterized such genetic elements in *E. coli* strains derived from host populations carrying both antibiotic resistance and metal resistance genes (Wyrsch et al., 2019; Zingali et al., 2020), these elements may also be present within the wastewater environment. Reflecting this, in an analysis of the plasmid metagenome from wastewater treatment plants, Li et al. (2015) identified a broad spectrum of both antibiotic and metal resistance genes. While several plasmids harboring antibiotic resistance genes or metal resistance genes were characterized, one plasmid was found to harbor both kinds of resistance genes concurrently. Alternatively, cross-resistance can occur through the broad-spectrum activity of resistance mechanisms conferring protection against multiple hazardous substrates at a time. Multi-drug efflux pumps, for instance, appear to mediate the efflux of toxic compounds in general, which includes antibiotics but can also apply to biocides and heavy metals (Blanco et al., 2016). As such, the harsh conditions of the wastewater matrix may be particularly conducive for the selection of antibiotic resistance in the wastewater microbiome.

The treatment processes employed during wastewater treatment may also select for antibiotic resistance, particularly in those microbial populations that are adept at surviving or even growing in this matrix. For instance, UV irradiation has been shown to activate the SOS response (Aksenov, 1999), which can promote the mobilization of resistance genes throughout the surviving population. Furthermore, chlorine disinfection has been shown to promote the transfer of resistance genes across microbial genera by enhancing the natural competency (i.e., uptake of naked DNA) of cells for mobile genetic elements that can transform the surviving population (Jin et al., 2020). As such, the wastewater treatment plant appears to be a particularly conducive environment for the selection of antibiotic resistance. Importantly, although studies have yet to identify distinct selection forces operating in wastewater treatment plants (Bengtsson-Palme et al., 2016), the selection of antibiotic resistance genes during treatment has been observed (Liu et al., 2018; Pazda et al., 2020), reinforcing the idea that wastewater treatment plants represent important hotspots for the evolution and selection of antibiotic resistance.

## Naturalized Wastewater Microbes and the Co-evolution of Antibiotic and Water Treatment Resistance

Characterization of the resistomes found in wastewater treatment plants suggest that naturalized microbes adapted to wastewater matrices may represent an important source of resistance genes contributing to the dissemination of antibiotic resistance in the microbial world. Unfortunately, the majority of the research has focused on clinical microbes in wastewater, even though clinical subpopulations are only transiently present in wastewater matrices due to their removal during the course of water treatment (Lu et al., 2015). In contrast, naturalized wastewater microbes may represent a persistent source of resistance genes driving the evolution of antibiotic resistance within the wastewater treatment plant.

### Characterization of Naturalized Wastewater *Escherichia coli* as a Model for the Co-evolution of Antibiotic and Water Treatment Resistance

A series of studies conducted by Zhi et al. (2016a, 2017, 2019) have characterized novel clonal strains of *E. coli* that appear to have evolved to survive and replicate in wastewater and sewage environments as their primary niche. These findings point to an interesting prospect concerning the life history of naturalized microbes and their role in the evolution of antibiotic resistance. Typically, natural microbial populations are conventionally understood as “ancient” microbial lineages that predate the existence of human or animal hosts; however, the characterization of naturalized wastewater *E. coli* suggests that host-derived bacterial populations may also evolve toward the adaptation and colonization of newly engineered environments. Notably, naturalized wastewater strains of *E. coli* have been primarily isolated from wastewater treatment plants and appear to be globally distributed (Zhi et al., 2016a, 2019). Considering that large-scale engineered wastewater treatment systems are a relatively new technology for sanitary management (becoming widespread only in the last century), it can be argued these naturalized wastewater strains represent a recently emerged subpopulation of *E. coli* that evolved to exploit a new engineered environment for its growth and survival.

Genetically and phenotypically, these naturalized *E. coli* strains were found to be distinct from both commensal and pathogenic intestinal (enteric) *E. coli* and the cryptic *Escherichia* lineages (Zhi et al., 2017, 2019; Wang et al., 2020). These strains were originally isolated from sewage samples treated with chlorine at doses sufficient to inactivate 99.99% of the *E. coli* population in the sample (4 log_10_ reduction), and therefore represented the chlorine resistant population found in sewage. Using a novel bioinformatic, logic regression-based approach to search for unique DNA sequence biomarkers in intergenic regions that define host/niche specificity (Zhi et al., 2015, 2016b), Zhi et al. (2016a) identified naturalized wastewater strains harboring a unique single-nucleotide polymorphic (SNP) biomarker in the *csgBAC-csgDEFG* and *asnS-ompF* intergenic loci, which was not found in 780 strains of enteric *E. coli* from humans and animals or the cryptic *Escherichia* clades (Zhi et al., 2016a). Of the chlorine tolerant population collected from treated sewage, 82% of all isolates recovered possessed this intergenic SNP biomarker specific for wastewater. Furthermore, 75% of these intergenic SNP biomarker positive strains were also found to harbor a unique insertion element, *IS30*, located specifically within the *uspC–flhDC* intergenic locus (GenBank accession number: ON075843). Subsequent whole genome analysis revealed that many of the isolates appeared to be clonal, even though they were isolated from different treatment plants in Canada (Zhi et al., 2019), and clonal-relatedness even extended to wastewater strains found in the United States and Switzerland (Zhi et al., 2019). PCR assays targeting the *uspC–IS30–flhDC* marker could easily distinguish these naturalized strains from their enteric counterparts (Zhi et al., 2016a). Despite this, the naturalized strains were found to: (a) be biochemically similar to enteric *E. coli* according to phenotypic biochemical analyses (i.e., Vitek 2® Automated Bacterial Analysis); (b) display a typical growth morphology on selective media commonly used to identify *E. coli* in clinical and environmental laboratories; and (c) phylogenomically group with enteric *E. coli* strains, away from the cryptic *Escherichia* clades when *Escherichia albertii* was used as the outgroup (Zhi et al., 2016a, 2019). Interestingly, while many *E. coli* strains found in water environments are classically associated with phylogroup B1 (Berthe et al., 2013; Touchon et al., 2020; Nowicki et al., 2021), the naturalized wastewater strains formed a separate cluster within phylogroup A within two sequence types, ST399 and ST635 (Zhi et al., 2019), suggesting that these strains may have undergone an evolutionary divergence from enteric strains of *E. coli*.

Up until very recently, and across the vast publicly-available genome database on *E. coli*, the *uspC-IS-30-flhDC* marker was only ever observed in *E. coli* strains isolated from wastewater treatment plants. However, Yang et al. (2021) recently identified *E. coli* strains carrying this biomarker in naturalized *E. coli* strains found in meat processing plants in Canada. This finding raises the prospect that these strains may have evolved to become naturalized populations in engineered environments, particularly those environments that employ disinfection processes.

Importantly, naturalized wastewater strains are characterized by several remarkable phenotypic adaptations for surviving the wastewater treatment/disinfection processes. In comparison to enteric strains, naturalized wastewater *E. coli* were found to be phenotypically resistant to various physiochemical water treatment processes, displaying 100,000-fold greater resistance to chlorination, an enhanced capacity for biofilm formation, greater resistance to advanced oxidants, and an extreme heat-resistant phenotype (Zhi et al., 2016a, 2017; Wang et al., 2020). The degree of heat resistance observed in these naturalized wastewater strains was far greater than what had been described for other heat-resistant strains of *E. coli*, designating them “extremely” heat resistant (Wang et al., 2020). This extreme heat resistance phenotype was linked to a genomic island known as the *locus of heat resistance* (LHR; Wang et al., 2020). Since heat is often used as a disinfection strategy in meat packing plants and for sewage treatment (i.e., composting of biosolids), maintenance of the LHR could be an adaptive solution for these naturalized strains to survive these environments. In addition, the LHR was also found to concurrently confer resistance to chlorine and advanced oxidants (Wang et al., 2020), processes that are common in water treatment. In turn, resistance to chlorine and oxidants recapitulates the fact that chlorine-based disinfectants are often used for sanitizing surfaces in meat packing plants. Interestingly, the LHR is flanked by transposons and has been found in other strains of *E. coli* (Mercer et al., 2015, 2017), suggesting that microbes have evolved diverse strategies to deal with multiple stressors present in a given environment.

The genomes of naturalized wastewater *E. coli* strains, as well as those found in meat packing plants, were also shown to be enriched for various stress-resistance genes compared to their enteric counterparts, likely enhancing their survival against treatment processes (Zhi et al., 2019; Yang et al., 2021). Interestingly, a significant number of stress/repair genes appear to have been acquired through horizontal gene transfer and are not commonly found in enteric *E. coli* strains (Zhi et al., 2019). Moreover, the genomes of these naturalized strains contain an over-abundance of stress-resistance genes (and environmental adaptation genes) compared to enteric strains (Zhi et al., 2019), including those associated with:

- DNA repair systems linked to damage caused by UV irradiation (i.e., a common wastewater treatment practice);
- type I and III restriction-modification systems, which cleave foreign DNA and are likely critical for survival in a sewage/wastewater environment that is abundant in diverse bacteriophages and conjugative plasmids (Dupuis et al., 2013);
- various oxidoreductases/glutathione transferase systems associated with resistance to oxidative stress, and implicated in resistance against UV, chlorine, and hydrogen peroxide (Patridge and Ferry, 2006; Allocati et al., 2009; Khemiri et al., 2014; Lemire et al., 2017);
- toxin-antitoxin systems that modulate nutrient starvation and intra-species competition in population-dense environments with limited resources (Christensen et al., 2001, 2003);
- heteromeric transposase elements known to be important for environmental adaptation by promoting horizontal gene transfer and integration of genomic islands among bacteria (Peters et al., 2014); and
- heavy metal resistance genes that may promote survival in response to the high concentrations of the many heavy metals present in wastewater matrices (Östman et al., 2017; Feng et al., 2018).

**Figure 2:**
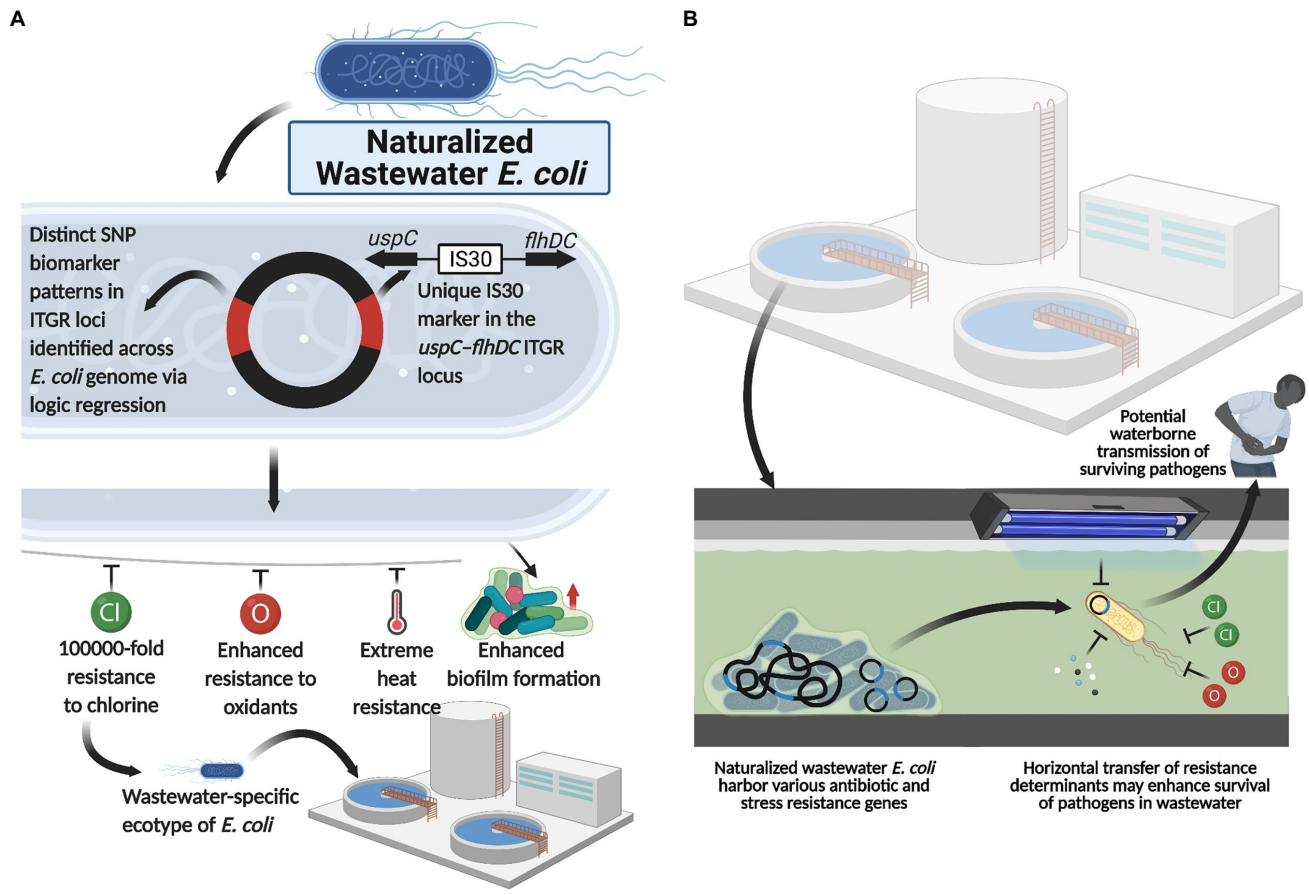
Naturalized wastewater *E. coli* as a reservoir for antibiotic and water treatment resistance. **(A)** Naturalized wastewater *E. coli* strains harboring unique SNP ITGR biomarkers and the *uspC–IS30–flhDC* genetic marker are characterized by various phenotypic adaptations, including extreme heat, chlorine and oxidant resistance as well as enhanced biofilm formation capacity, which appear to underlie their recent naturalization into wastewater matrices. In line with this, these naturalized strains harbor an abundance of antibiotic and stress resistance genes that may be mobilized and acquired by other microbes in the wastewater matrix, including pathogenic strains. Importantly, this suggests that **(B)** naturalized wastewater microbes may serve as reservoirs for both antibiotic resistance and water treatment resistance, enhancing the survival and transmission of pathogens present in wastewater. Created with BioRender.com.

Interestingly, functional genomics revealed that, compared to enteric *E. coli*, these naturalized wastewater strains simultaneously lacked various genes implicated in host colonization processes (i.e., adhesin synthesis genes) and pathogenesis (i.e., type IV secretion system genes; Zhi et al., 2019; Yang et al., 2021). Collectively, the genetic, phylogenomic, phenotypic, and ecotypic evidence suggest that naturalized wastewater-specific *E. coli* strains appear to have evolved to exploit wastewater/sewage treatment plants (and potentially meat packing plants) as their primary niche for growth and survival (Figure 2A), and concomitantly abandoned life in the gut of humans and animals. Indeed, their evolution from a host-colonizing to naturalized population is supported by phylogenomic analyses that demonstrate their evolutionary relationship to, and divergence from, enteric *E. coli* strains within phylogroup A (Zhi et al., 2019).

Interestingly, water treatment resistant naturalized *E. coli* also possessed a plethora of antibiotic resistance (AR) genes and for which a common backbone of 47 AR genes were shared across the genomes of all naturalized strains isolated from wastewater treatment plants in Canada, Switzerland, and the United States (Table 1). The global distribution of a common backbone of AR genes in naturalized strains suggests that these AR-genes may play an important and adaptive role in their survival in the wastewater matrix, possibly during secondary treatment (i.e., a stage of intense microbial competition, predation, etc.) or in response to contaminants such as heavy metals or other noxious compounds. Notably, the vast majority of this shared repertoire of AR genes include multi-drug efflux pumps (Table 1), which presumably mediate broad-spectrum activity against a plethora of hazardous substrates that may be present in wastewater (Blanco et al., 2016). Interestingly, despite possessing a plethora of antibiotic resistance genes, the strains themselves were shown to be susceptible to most antibiotics when tested at clinical breakpoints (Zhi et al., 2019). As such, the specific roles that these resistance genes play in the life history and survival of the naturalized wastewater strains remains to be determined, but could involve endogenous resistance to bioactive molecules from other microbes, hormesis, quorum sensing, and/or intercellular signaling (as discussed in the previous section of this review). Importantly, several studies have revealed a possible link between antibiotic resistance and water treatment resistance. For instance, Huang et al. (2013) demonstrated a phenotypic relationship between chlorine resistance and tetracycline resistance, suggesting that chlorine treatment may promote the selection of *E. coli* isolates highly resistant to tetracycline. Additionally, Liu et al. (2018), demonstrated that the use of chlorine dioxide during water treatment resulted in the selection of macrolide (*ermB*), tetracycline (*tetA*, *tetB*, and *tetC*), sulfonamide (*sul1*, *sul2*, and *sul3*), β-lactam (*ampC*), aminoglycoside [*aph(2′)-Id*], rifampicin (*katG*), and vancomycin (*vanA*) resistance genes in treated wastewater effluent samples. Interestingly, three of these genes, including *ermB*, *ampC*, and *sul1*, were harbored by the naturalized wastewater *E. coli* strains described by Zhi et al. (2019). Collectively, these data suggest that AR genes in naturalized strains may enhance survival against specific wastewater treatment processes, which can lead to their enrichment across the treatment process (Laht et al., 2014; Mao et al., 2015; Bengtsson-Palme et al., 2016). Alternatively, as these strains appear to be adapted to wastewater matrices, their endogenous growth can lead to the amplification of harbored AR genes within the wastewater matrix—such that these strains act as a persistent reservoir of genetic elements that may simultaneously confer antibiotic resistance and promote survival within wastewater (Figure 2B).

**Table 1:** A list of the common suite of antibiotic resistance genes found in naturalized wastewater *Escherichia coli* strains (based on Zhi et al., 2019) implicating their importance in promoting survival and resistance to wastewater treatment and a comparison of the carriage of these genes in water treatment resistant extra-intestinal pathogenic *Escherichia coli* isolated from wastewater (based on Zhi et al., 2020).^a^

Antibiotic resistance gene in naturalized wastewater *Escherichia coli*^b^	Resistance mechanism	Drug or drug class	Percentage of urinary pathogenic *Escherichia coli* (*n* = 24) isolated from wastewater and that carry the specified AR gene (%)^c^
acrA	Efflux pump	Triclosan, glycylcycline, phenicol, penam, cephalosporin, tetracycline, fluoroquinolone, and rifamycin	100
acrB	Efflux pump	Triclosan, glycylcycline, phenicol, penam, cephalosporin, tetracycline, fluoroquinolone, and rifamycin	100
acrD	Efflux pump	Aminoglycosides	5
acrE	Efflux pump	Fluoroquinolone, cephalosporin, penam, and cephamycin	100
acrF	Efflux pump	Fluoroquinolone, cephalosporin, penam, and cephamycin	100
acrR	Efflux pump, antibiotic target alteration/replacement	Triclosan, glycylcycline, phenicol, penam, cephalosporin, tetracycline, fluoroquinolone, and rifamycin	100
acrS	Efflux pump	Triclosan, glycylcycline, cephalosporin, penam, cephamycin, tetracycline, fluoroquinolone, rifamycin, and phenicol	100
ampC beta-lactamase	Antibiotic inactivation	Cephalosporin and penam	100
arnA	Antibiotic target alteration/replacement	Peptide antibiotics	-
bacA	Antibiotic target alteration/replacement	Peptide antibiotics	100
baeR	Efflux pump	Aminocoumarin and aminoglycoside	96
baeS	Efflux pump	Aminocoumarin and aminoglycoside	96
cpxA	Efflux pump	Aminocoumarin and aminoglycoside	100
CRP	Efflux pump	Macrolide, fluoroquinolone, and penam	100
emrA	Efflux pump	Fluoroquinolone	100
emrD	Efflux pump	Meta-chlorocarbonylcyanide phenylhydrazone, linezolid, rifampicin, erythromycin, trimehtoprim, and chloramphenicol	-
emrK	Efflux pump	Tetracycline	100
emrR	Efflux pump	Fluoroquinolone	100
emrY	Efflux pump	Tetracycline	100
evgA	Efflux pump	Macrolide, penam, tetracycline, and fluoroquinolone	100
evgS	Efflux pump	Macrolide, penam, tetracycline, and fluoroquinolone	100
GlpT	Reduced membrane permeability	Fosfomycin	96
H-NS	Efflux pump	Macrolide, penam, cephamycin, tetracycline, cephalosporin, and fluoroquinolone	100
kdpE	Efflux pump	Aminoglycoside	100
marA	Efflux pump, reduced membrane permeability	Triclosan, glycylcycline, cephalosporin, penam, cephamycin, monobactam, penem, tetracycline, fluoroquinolone, rifamycin, phenicol, and carbapenem	100
marR	Efflux pump	Triclosan, glycylcycline, phenicols, penam, cephalosporin, tetracycline, fluoroquinolone, and rifamycin	100
mdfA	Efflux pump	Rhodamine, benzalkonium chloride, and tetracycline	100
mdtA	Efflux pump	Aminocoumarin	100
mdtB	Efflux pump	Aminocoumarin	100
mdtC	Efflux pump	Aminocoumarin	96
mdtE	Efflux pump	Macrolide, fluoroquinolone, and penam	100
mdtF	Efflux pump	Macrolide, fluoroquinolone, and penam	100
mdtG	Efflux pump	Fosfomycin	100
mdtH	Efflux pump	Fluoroquinolone	100
mdtM	Efflux pump	Nucleoside, lincosamide, fluoroquinolone, acridine dye, and phenicol	54
mdtN	Efflux pump	Nucleoside antibiotics and acridine dye	96
mdtO	Efflux pump	Nucleoside antibiotics and acridine dye	100
mdtP	Efflux pump	Nucleoside antibiotics and acridine dye	100
msbA	Efflux pump	Nitroimidazole	100
msrB	Efflux pump	Streptogramin and macrolide	0
patA	Efflux pump	Fluoroquinolone	0
PmrC	Antibiotic target alteration/replacement	Peptide antibiotics	0
PmrF	Antibiotic target alteration/replacement	Peptide antibiotics	100
soxR	Efflux pump, antibiotic target alteration/replacement	Triclosan, glycylcycline, cephalosporin, penam, tetracycline, fluoroquinolone, rifamycin, and phenicols	100
soxS	Efflux pump, antibiotic target alteration/replacement, reduced membrane permeability	Triclosan, glycylcycline, cephalosporin, penam, cephamycin, carbapenem, monobactam, penem, tetracycline, fluoroquinolone, rifamycin, and phenicol	100
TolC	Efflux pump	Triclosan, macrolide, penam, cephamycin, aminocoumarin, tetracycline, glycylcycline, cephalosporin, fluoroquinolone, rifamycin, and phenicol	100
YojI	Efflux pump	Peptide antibiotics	100

a The dataset is based on whole genome sequences from naturalized wastewater *Escherichia coli* strains and UPEC strains and bioinformatically analyzed through the Canadian Antimicrobial Resistance Database (CARD), methods of which are described by Zhi et al. (2019, 2020).

b Genes are inclusive of naturalized wastewater *Escherichia coli* strains possessing the SNP biomarker as described by Zhi et al. (2016a) and include strains from Canada, Switzerland, and the United States (Zhi et al., 2019).

c Gene analysis is based whole genomes sequenced from of a library of 24 UPEC isolates collected from sewage/wastewater (Zhi et al., 2020).

Beyond their intrinsic resistance genes, recent data also suggests that naturalized wastewater *E. coli* may be permissive and competent at acquiring antibiotic resistance genes from clinical strains. For example, an *E. coli* isolate collected from a hospital wastewater system in Switzerland by Zurfluh et al. (2017a, b), which was subsequently shown to be a naturalized wastewater strain by Zhi et al. (2019), possessed various carbapenem resistance genes, including a plasmid-encoded 16S rRNA methyltransferase gene (*rmtB*) and the β-lactamase *bla*_TEM-1b_—common constituents in extended spectrum β-lactamase (ESBL) clinical isolates of *E. coli*. Interestingly, naturalized wastewater strains collected in other studies lacked these particular genes (Zhi et al., 2019); however, strains from Zhi et al. (2019) were isolated from municipal sewage, whereas Zurfluh et al. (2017b) isolated their naturalized *E. coli* strain directly from a hospital sewage/wastewater system. Considering that antibiotic residues in untreated hospital wastewater are much greater than those typically seen in municipal wastewater (Karthikeyan and Meyer, 2006), heightened exposure to antibiotics in hospital wastewater may provide the necessary selective pressure for the retention of some AR genes (i.e., carbapenem resistance genes and beta-lactams) in naturalized wastewater *E. coli* strains (Zurfluh et al., 2017b). As such, there is evidence to suggest that naturalized wastewater *E. coli* strains may be amenable to the acquisition and transfer of resistance genes, including the possibility of acquiring conjugative plasmids from enteric strains and/or the uptake of free AR genes from the wastewater matrix (i.e., transformation).

The abundance of AR genes and the resistance of naturalized wastewater *E. coli* strains to water treatment is cause for concern for several reasons. Firstly, although naturalized wastewater *E. coli* isolates may *not* be a direct threat to humans (i.e., not capable of infecting humans given their specificity for wastewater), they represent an important and persistent reservoir of AR genes *and* water treatment resistance genes. Secondly, sewage/wastewater may act as an amplification matrix for AR and water treatment resistance genes through the endogenous growth and replication of these naturalized strains within these matrices. Thirdly, there exists the possibility of horizontal gene transfer of AR genes and possibly water treatment resistance genes from naturalized wastewater microbes to environmental microbes, a phenomenon that has been previously demonstrated (Marcinek et al., 1998; Karkman et al., 2018). Fourthly, and most importantly, since naturalized wastewater *E. coli* appear to display some resistance to water treatment, and for which resistance might be related to AR-genes or other transmissible elements (e.g., the LHR), naturalized strains represent an important genetic reservoir for the potential emergence of pathogenic *E. coli* resistant to both water treatment and antibiotics—a concerning prospect considering that water treatment and sanitation represent the greatest human achievements for the control of infectious disease in modern society.

### The Role of Naturalized Wastewater *Escherichia coli* in the Emergence of Antibiotic and Water Treatment Resistance in Extraintestinal Pathogenic *Escherichia coli*

A growing body of evidence also points to the possibility that naturalized wastewater *E. coli* strains may be playing an important role in the emergence of certain pathotypes of *E. coli* in wastewater. Reflecting this, several studies have demonstrated that extraintestinal pathogenic subpopulations of *E. coli* appear to be differentially capable of surviving wastewater treatment (Table 2). For instance, Anastasi et al. (2013), observed that various strains of pathogenic *E. coli* differentially survive wastewater treatment processes, and notably, the surviving population was predominated by ExPEC pathotypes, in particular, the urinary pathogenic *E. coli* (UPEC). In another study, Anastasi et al. (2010) observed that almost 60% of isolates surviving wastewater treatment possessed one or more virulence genes associated with the UPEC pathotype (*papA/H*, *papE/F*, *papC*, *hlyA*, *cnf1*, and *iroN*). Similarly, using a virulence gene profiling approach, Adefisoye and Okoh (2016) observed that >41.7% of *E. coli* isolates from treated wastewater effluents represented potential UPEC strains. Likewise, Calhau et al. (2015), identified UPEC in wastewater effluents based on the presence of UPEC-associated virulence genes (i.e., *iutA*, *papA/H*, and *sfa*) and pathogenicity islands (i.e., PAI IV_536_, PAI I_CFT073_, PAI II_CFT073_), leading to the observation that UPEC strains dominated the *E. coli* population in hospital treated wastewaters, and included extended spectrum beta-lactamase (ESBL) producing strains belonging to the pandemic-associated sequence type (ST) 131 lineage.

**Table 2:** Summary of studies evaluating the prevalence of extraintestinal pathogenic *Escherichia coli* (ExPEC) in treated wastewater.

References	Proportion of ExPEC in wastewater effluent	Method used for identification of isolate
Anastasi et al., 2010	264 *Escherichia coli* isolates from four sewage treatment plants: 59.5% carried at least one UPEC virulence gene (*papAH*, *papEF*, *papC*, *hlyA*, *cnf1*, and *iroN*). None of the isolates carried enteric pathotype genes (*eltA*, *estII*, *ipaH*, *eaeA*, *stx1*, *stx2*).	Virulence Gene Screening
Anastasi et al., 2013	370 *Escherichia coli* wastewater isolates collected across wastewater treatment: Five of seven biochemical phenotypes (i.e., lineages) surviving UV radiation were found to carry UPEC virulence genes (*papAH*, *papEF*, *papC*, *hlyA*, *cnf1*, *iroN*, *chuA*, and *yjaA*). Only one isolate carried virulence genes that were associated with intestinal pathogenic *Escherichia coli* strains (*eltA*, *estII*, *ipaH*, *eaeA*, *stx1*, and *stx2*).	Virulence Gene Screening, Biochemical Fingerprinting/Phenotyping
Calhau et al., 2015	193 *Escherichia coli* isolates from hospital and municipal wastewater: 20 unique fingerprinting patterns were identified and of the representative isolates chosen for each rep-PCR pattern, 15 patterns carried UPEC-associated genes (*papAG*, *papC*, *sfa/foc*, *ada/dra*, *iutA*, and *kpsMII*) and/or pathogenicity islands (PAI I_536_, PAI II_536,_ PAI III_536_, PAI IV_536_, PAI I_J96_, PAI II_J96_, PAI I_CFT073_, and PAI II_CFT073_).	rep-PCR Fingerprinting and Virulence Gene/Pathogenicity Island Screening
Zhi et al., 2020	376 *Escherichia coli* isolates recovered from chlorinated sewage: None possessed the *stx1/2* gene (associated with STEC). 93 isolates possessed at least one UPEC virulence gene (*papC*, *sfa/foc*, *fyuA*, *chuA*, and *iroN*). 24 of the 93 possessed at least three UPEC virulence genes. 14 of the 24 were found to share high (≥96.03%) whole genome similarity with a confirmed clinical UPEC strains.	Virulence Gene Screening, Pairwise Whole Genome Alignment
Adefisoye and Okoh, 2016	223 *Escherichia coli* isolates recovered from finished wastewater effluents: 41.7% represented potential UPEC (*papC*-positive). 14.8% represented potential neonatal meningitic *Escherichia coli* (NMEC; *ibeA*-positive). 7.6% represented potential enteroaggregative *Escherichia coli* (EAEC; *eagg*-positive). 7.6% represented potential enteropathogenic *Escherichia coli* (EPEC; *eae*-positive). 1.4% represented potential enterotoxigenic *Escherichia coli* (ETEC; *lt 3*-positive).	Virulence Gene Screening
Mokracka et al., 2011	109 *Escherichia coli* isolates recovered from wastewater: 50.5% represented potential ExPEC (*fyuA*, *iutA*, *hlyA*, *papA*, *sfaS*, *focG*, and *cnf1*) 20.2% were characterized as potential diarrheagenic strains including ETEC (LT/ST genes), Shiga toxin-producing *Escherichia coli* (STEC) [*stx1/2*], EPEC (*eae* and *bfpA*), EAEC (CVD432), and EIEC (*ipaH*)	Virulence Gene Screening and Phylogenetic Analysis
Diallo et al., 2013	416 *Escherichia coli* isolates collected from treated municipal wastewater effluent: 27 carried at least one ExPEC associated genetic marker (*papEF*, *sfa/focDE*, *afa/draBC*, *cnf*, *f17A*, *hlyA*, *clbN*, and *kpsMT K1*), of which 21 were characterized as potential ExPEC; two were characterized as atypical EPEC (*eae*); and none were characterized as STEC (*stx*).	Virulence Gene Screening
Frigon et al., 2013	719 *Escherichia coli* isolates collected from four wastewater treatment plants: 24% of all isolates represented potential ExPEC; 10% were characterized as intestinal pathotypes; 6% carried virulence genes correlating with both ExPEC and intestinal pathogenic pathotypes (based on over 100 virulence genes monitored or used directly for pathotyping).	Virulence Gene Screening
Franz et al., 2015	82 *Escherichia coli* isolates collected from wastewater: 11.4% were characterized as ExPEC based on the presence of at least three ExPEC associated virulence genes (*afa*, *focG*, *hlyD*, *iutA*, *kpsMII*, *papA*, and *sfaS*), whereas 7.8% were described as potential diarrheagenic strains (*stx1/2*, *eae*, *aggR*, *ipaH*, *estA*, and *eltB*).	Virulence Gene Screening
Gomi et al., 2017	54 *Escherichia coli* isolates were collected from wastewater treatment plants and hospitals and chosen for genome sequencing: 44.4% were identified as ExPEC based on the presence of ExPEC-associated virulence genes.	Virulence Gene Screening and Whole Genome Sequencing
Mahfouz et al., 2018	92 *Escherichia coli* isolates recovered from treated wastewater: Eight were identified as potential ExPEC (harboring at least 20 of 58 screened ExPEC virulence genes).	Virulence Gene Screening and Whole Genome Sequencing
Savin et al., 2021	71 *Escherichia coli* isolates recovered from wastewater collected from poultry and pig slaughterhouses: 17.1% and 5.6% were characterized as ExPEC, respectively, according to the presence of *iutA* and *kpsMII*.	Virulence Gene Screening

In line with these findings, a comprehensive genomic analysis performed by Zhi et al. (2020) identified UPEC strains as common constituents within treated sewage and wastewater effluents, and *E. coli* strains recovered from wastewater matrices were found to cluster within several clinically relevant sequence types including ST131, ST95, ST127, and ST640. Several wastewater strains were found to be virtually identical to clinical UPEC strains across the whole genomes (i.e., as high as 99.49% genome similarity), and many carried the exact same complement of virulence genes as their clinical counterparts. Concerningly, the wastewater-derived UPEC isolates also carried a plethora of antibiotic resistance genes, and several isolates were identified as belonging to the pandemic O25b-ST131 clonal group representing potential ESBL-producing strains. The remarkable similarity between the wastewater isolates and clinical UPEC strains, coupled with the findings of others, led the authors to conclude that UPEC strains possess a differential capacity to survive full scale wastewater treatment (Zhi et al., 2020). While these findings do not discount the survival of intestinal commensal and pathogenic *E. coli* during water treatment (Omar and Barnard, 2014), the evidence seems to indicate that ExPEC strains are disproportionately represented in the surviving population of *E. coli* in wastewater (Table 2), and which has led some authors to suggest that “higher-than-expected” levels of ExPEC infection may exist in the community or that ExPEC strains naturally occur in wastewater (Paulshus et al., 2019).

Interestingly, several lines of evidence point toward a close relationship between naturalized wastewater *E. coli* strains and emerging water treatment-resistant ExPEC populations. As previously outlined, all naturalized wastewater *E. coli* strains share a common complement of antibiotic resistant genes (Table 1), potentially implying their importance in mediating survival in a wastewater matrix (Zhi et al., 2019). Remarkably, UPEC strains also possess many of these antibiotic resistance genes (Table 1), suggesting some common mechanisms that may underlie the differential survival of UPEC and naturalized strains during wastewater treatment. Although naturalized wastewater *E. coli* strains appear to lack certain genes associated with adhesion/colonization and pathogenesis in the gut, they possessed upward of 36 different UPEC-related virulence genes, including: (a) the ferrienterobactin multi-enzyme sythetase complex and transport system; (b) various UPEC fimbrial genes; (c) the curli virulence gene (*csgB*); (d) the *ompA* outer membrane protein gene; and (e) the flagellar biosynthesis protein encoded by *flhA* (Zhi et al., 2019). Some naturalized strains also possessed the yersiniabactin siderophore biosynthetic pathway and receptor/transport system found in many clinical UPEC, and some strains also possessed the UPEC virulence genes *fyuA* and *irp*1/2 (Zhi et al., 2019). It is interesting to note that the virulence genes highlighted above have been identified as being important in the survival of UPEC in the *extraintestinal* environment, particularly in the nutrient-deprived and osmotically-stressed environment of the urinary tract characterized by low solute concentrations and repetitive flushing (Mann et al., 2017). Many of the shared virulence genes between UPEC and naturalized strains are iron-sequestering genes (e.g., ferribactin, yersiniabactin, and enterobactin-related genes and transport pathways), and known to be important for UPEC’s survival in iron-deficient urine (Terlizzi et al., 2017). Considering that wastewater is similarly characterized by a limited availability of iron at dilute concentrations, these genes may play a similar role for the naturalized strains in the wastewater environment. Notably, many of these so-called UPEC-related virulence genes were also found in the genomes of environmental cryptic clades of *Escherichia* (Zhi et al., 2019), providing further support for the idea that the primary function of these genes may be for survival in environmental niches (i.e., urine, water, sewage, and wastewater). It is also interesting to note that naturalized wastewater strains possess the UPEC-associated virulence gene *irp*, and this gene has been linked to protection against protozoan grazing and predation (Adiba et al., 2010). Consequently, these so-called “UPEC virulence genes” may actually represent environmental adaptation genes necessary for the survival of *E. coli* outside of the intestinal environment.

There is also growing speculation that water may be an important, and underestimated, vehicle of transmission for ExPEC (Graham et al., 2021). Indeed, urinary tract infections, the vast majority of which are caused by UPEC, have been epidemiologically-linked to exposures associated with contaminated recreational water (Søraas et al., 2013). Similarly, in a study by Tanner et al. (2019), ESBL-producing Enterobacteriaceae, including *E. coli*, were found in 6.4% of treated drinking water samples that failed bacteriological water quality parameters in the United States (i.e., total coliforms), leading the authors to conclude that treated drinking water may be an underestimated vehicle for transmission of ExPEC in the community.

Other than the indirect evidence provided above that sheds some light on the phenotypic (i.e., ability to survive wastewater treatment) and genotypic relationship (i.e., shared AR and virulence genes) between naturalized wastewater strains of *E. coli* and ExPEC, there is no definitive evidence (as of yet) that clearly demonstrates genetic compatibility between naturalized *E. coli* and ExPEC strains and the transfer of AR or water treatment-resistant genes between these populations. Nevertheless, and as demonstrated throughout this paper, wastewater is known to be an environmental “hotspot” for AR bacteria, AR genes and the selection forces that drive evolutionary emergence of antibiotic resistance. The story of naturalized wastewater *E. coli* and the potential emergence of water treatment and antibiotic resistance in pathogenic populations is concerning, especially considering that *E. coli* is only one species present in a vast wastewater microbiome. Indeed, the very same natural selection forces could be driving the naturalization of other microbial populations in wastewater and subsequent evolution of water treatment resistance in other pathogenic populations, such as the ESKAPE pathogens. Clearly, the impact to public health could be substantial and more research is needed.

## Conclusion

The widespread prevalence of antibiotic resistance in the microbial world represents one of the most critical problems facing public health today; however, the genetic determinants underlying the current resistance crisis appear to have their origins in ancient, naturalized microbes, indicating that naturalized microbial populations have played key roles in the origin, dissemination, and evolution of antibiotic resistance. The recent discovery of naturalized wastewater-specific strains of *E. coli* bring to light an even more ominous specter that antibiotic resistance could be linked to water treatment resistance. Importantly, water treatment represents the single greatest public health intervention for control of infectious diseases in modern society, and the idea that microbes may be breaching this barrier, is very concerning, and more research investment and policy action is needed in this area.

## Author Contributions

DY and NN wrote the bulk of the manuscript with scientific and editorial insights from SO, SZ, and KR. DY, SZ, and KR generated research data regarding naturalized wastewater *E. coli*. SO and NN provided the funding support. All authors contributed to the article and approved the submitted version.

## Funding

Funding was provided by a grant to SO and NN from the Albert Ministry of Jobs, Economy, and Innovation (Major Innovation Fund) for the Antimicrobial Resistance – One Health Consortium, the Canadian Institutes for Health Research (CIHR; to NN), the Natural Sciences and Engineering Research Council (NSERC; to NN), Alberta Innovates (to NN and SO), National Natural Sciences Foundation of China (grant number: 82073514), and Ningbo Natural Sciences Foundation (grant number: 202003N4114).

## Conflict of Interest

The authors declare that the research was conducted in the absence of any commercial or financial relationships that could be construed as a potential conflict of interest.

## Publisher’s Note

All claims expressed in this article are solely those of the authors and do not necessarily represent those of their affiliated organizations, or those of the publisher, the editors and the reviewers. Any product that may be evaluated in this article, or claim that may be made by its manufacturer, is not guaranteed or endorsed by the publisher.
